# Human T Cell Memory: A Dynamic View

**DOI:** 10.3390/vaccines5010005

**Published:** 2017-02-04

**Authors:** Derek C. Macallan, José A. M. Borghans, Becca Asquith

**Affiliations:** 1 Institute for Infection & Immunity, St George’s, University of London, Cranmer Terrace, London SW17 0RE, UK; 2 St George’s University Hospitals NHS Foundation Trust, Blackshaw Road, London SW17 0QT, UK; 3 Laboratory of Translational Immunology, Department of Immunology, University Medical Center Utrecht, P.O. Box 85090, 3508 AB Utrecht, The Netherlands; j.borghans@umcutrecht.nl; 4 Department of Medicine, Imperial College London W2 1PG, UK; r.asquith@ic.ac.uk

**Keywords:** immune memory, kinetics, dynamics, vaccine, vaccination, proliferation, turnover, survival

## Abstract

Long-term T cell-mediated protection depends upon the formation of a pool of memory cells to protect against future pathogen challenge. In this review we argue that looking at T cell memory from a dynamic viewpoint can help in understanding how memory populations are maintained following pathogen exposure or vaccination. For example, a dynamic view resolves the apparent paradox between the relatively short lifespans of individual memory cells and very long-lived immunological memory by focussing on the persistence of clonal populations, rather than individual cells. Clonal survival is achieved by balancing proliferation, death and differentiation rates within and between identifiable phenotypic pools; such pools correspond broadly to sequential stages in the linear differentiation pathway. Each pool has its own characteristic kinetics, but only when considered as a population; single cells exhibit considerable heterogeneity. In humans, we tend to concentrate on circulating cells, but memory T cells in non-lymphoid tissues and bone marrow are increasingly recognised as critical for immune defence; their kinetics, however, remain largely unexplored. Considering vaccination from this viewpoint shifts the focus from the size of the primary response to the survival of the clone and enables identification of critical system pinch-points and opportunities to improve vaccine efficacy.

## 1. Introduction

The introduction of vaccines has had a revolutionary effect on human health and life expectancy over the last 50 years. Despite these huge advances, many basic immunological questions about how long-term immunological memory is maintained remain unanswered. While many successful vaccines act primarily by generating antibodies, there is also a clear need for vaccines that generate populations of highly-specific T cells, especially against infectious agents that successfully escape antibody responses.

At its most basic mechanistic level, T cell vaccine efficacy can be defined as the ability of the vaccine to generate large, effective, long-lived populations of memory T cells. In this paper we will take a dynamic view of how long-term T cell memory may be generated and maintained in humans and review the implications of a kinetic perspective for how we think about vaccine responses.

## 2. Part I: Thinking Kinetically—How Long Is a Memory?

### 2.1. The Longevity of Immune Responses

The key desirable property of a vaccine response (or the desirable consequence of natural-immunity) is longevity. Duration of protection varies widely from one vaccine to another, and from one pathogen to another. However, for many vaccines and infections, immunity is remarkably long-lived—indeed, frequently life-long. The yellow fever vaccine represents a good example; it now appears that a single dose confers life-long immunity [1]. Similarly, influenza immunity can last for decades. Very elderly people appeared to be protected from the 2009 H1N1 “swine flu” influenza epidemic [2] by previous exposure to a structurally-similar strain which circulated widely following the 1918 epidemic [3]. There may be differences in longevity between B-cell and T cell immunity. For example, for smallpox, post-vaccination B-cell memory was found to be stable for more than 50 years, whereas CD4^+^ and CD8^+^ T cell memory declined slowly with a half-life of 8–15 years [4,5]. Nonetheless, the take-home message is the same: immune memory is remarkably long-lived.

### 2.2. The Paradox of Long-Lived Memory and Short-Lived Cells

How is such longevity achieved? The oft-repeated view is that long-term immune memory results from the generation of “memory” cells, which intrinsically and individually have a long lifespan. Such cells are often referred to as “*long-lived memory-cells*”, the emphasis being on the longevity of the cells, rather than the memory. The problem with this paradigm is that it conflicts with a large body of experimental data, all of which suggest that memory T cells have limited lifespans, certainly considerably shorter than the lifespan of immunological memory itself. For both CD4^+^ and CD8^+^ T cells, although memory T cell populations persist, the individual cells that comprise those populations proliferate and die at a non-insignificant rate [6]. For B-cells, a similar pattern is seen, memory cells having a faster turnover rate than naive cells, but there is a significant contrast in that B cells provide long-term protection thanks to their progeny—long-lived plasma cells [7].

This concept of rapid memory cell turnover is supported by several lines of experimental evidence. Already 25 years ago, it was imputed from the disappearance rates of CD45R0^+^ T cells with chromosome damage [8]. More recently, studies with Ki67 staining have shown that memory T cells have higher proliferation rates than naive T cells [9,10]. Finally, direct measurement of cellular lifespans using in vivo stable isotope labelling has shown that memory T cells are much shorter-lived than their naive counterparts in both mice and men, in both young and old, and are also much shorter-lived than the immunological memory they convey [11,12,13,14,15]. The lifespan of a human memory T cell is of the order of 30–160 days [12,13,15,16], in contrast to the typical half-life of human T cell memory of 8–15 years [1,4,5]. Longevity thus does not seem to be an intrinsic characteristic of circulating memory T cells. 

### 2.3. Resolving the Paradox—Short-Lived Cells Conferring Long Lived-Memory

One way to resolve this apparent paradox is to view cellular behaviour from a kinetic viewpoint. As Michie et al. observed in 1992, “… *memory is maintained by long-lived clones rather than individual cells with a long lifespan*” [17]. The pivotal concept here is that it is the *population* that must be long-lived, not necessarily the individual *cell*. Hence, we need to refer to “*cells conferring long-lived-memory*”, rather than “*long-lived memory cells*”. This concept is widely accepted and very familiar to people studying the dynamics of T cell memory but has been slow to be adopted into some areas of immunology.

**Figure 1:**
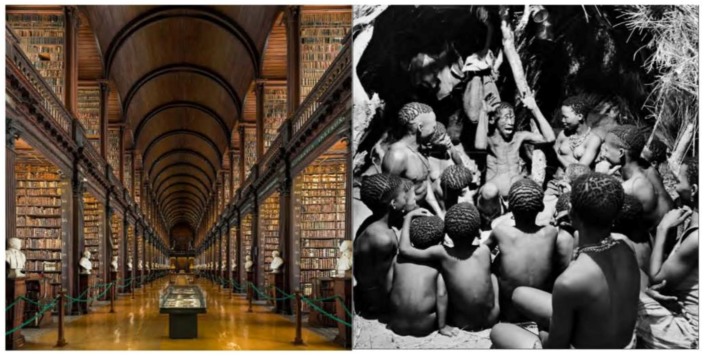
Contrasting ways to store memories—the archive and the story teller. On the left, the Library at Trinity College Dublin (Photo by DAVID ILIFF. License: CC-BY-SA 3.0); the right-hand frame shows a traditional story-teller or Griot, describing, perhaps, what to do on encountering cognate antigen (Photograph: Bushmen, N. R. Farbman for Life magazine, 1946; http://images.google.com/hosted/life/6da6b5c69e36d1ff.html; © Time Inc).

The contrast between the static and kinetic view of memory can be illustrated by an example from the history of the transfer of knowledge (Figure 1). When Ptolemy I Soter I wanted to capture the current knowledge of the world in the 4th century BCE, he built the great library of Alexandria in which the finest manuscripts of the day were stored. The memory was static; once the scroll was stored, the information on it remained captured and immutable, but accessible for recall at a later date. This is analogous to the static view of anamnestic immunity—in which memory is preserved in a population of long-lived cells, available for recall when needed. An alternative view is provided by African tribal elders who preserve their communal memory by entrusting their truths to story-tellers and singers, the *Griots*, who sing the stories of the tribe and pass them on to future generations. The story is dynamic and can be retold in different contexts, but maintains its core elements through each retelling. A dynamic model of immune memory may be more like the latter—stories of prior vaccine or pathogen exposure being retold in the clonal replication of memory cells from one cellular generation to another.

### 2.4. Are There Really No Long-Lived Memory-Cells?

Of course, this does not exclude the possibility that some T cells really are long-lived. Two other scenarios deserve consideration, and neither is mutually exclusive with the kinetic model. The first is that there are long-lived memory T cells in the circulation—it is just that we have not identified them yet; the second is that long-lived memory cells exist, but occur primarily outside the circulation.

### 2.5. Circulating Memory Cells

In terms of circulating cells, in vivo stable isotope labelling experiments analyse events in bulk populations and hence theoretically may miss a relatively small subpopulation of long-lived cells masked by a majority of dividing cells. Interestingly, no studies have yet identified a phenotype that corresponds to a circulating long-lived memory T cell. It would be surprising if there were a subpopulation of long-lived cells with no such marker, although it is possible that the only markers are transcriptomic or epigenetic, rather than phenotypic [18]. 

### 2.6. Tissue-Resident Memory

The second scenario posits that long-term memory T cells do exist, but primarily reside outside the circulation. This is difficult to test using human in vivo data as most such studies rely on sampling blood. Although lymph-node T cells recirculate [19], other T cell populations, such as those in bone marrow or in non-lymphoid tissues may act as long-term resident memory. Recent studies have shown that there is a substantial pool of memory T cells in diverse non-lymphoid tissues, including skin, intestine and brain. These cells, which mostly express CD69, have a tendency to stay locally and not to recirculate, and have therefore been coined tissue-resident memory T cells (T_RM_) [20,21,22]. T_RM_ cells are thought to be very important for efficient clearance of recurring infections at the port of pathogen entry, and appear functionally superior to circulating memory T cells [20,21]. New vaccine strategies are currently being developed to trigger such T_RM_ responses, with promising results [20,23]. Very little is known, however, about the mechanism by which such T_RM_ cells are maintained. Since their virtual absence in peripheral blood hampers the study of their turnover, one cannot exclude the possibility that they reside as long-lived memory cells at multiple non-lymphoid locations. 

Bone marrow also contains memory T cells (perhaps analogous to B-cell derived plasma cells). In contrast to the anticipated role of T_RM_ in other tissues—to provide local protection at sites of pathogen entry—such bone-marrow memory T cells (T_BM_) are thought to contribute to systemic memory [24]. Interestingly, in many respects the phenotype of T_BM_ resembles that of T_RM_ cells, with relatively high expression of CD69, and low expression of S1P_1_ and Klf2 [25,26]. Although some evidence implies that T_BM_ may be genuinely long-lived and may thus represent an archive-like repository of memory [25,27], other data suggest that the vast majority of T_BM_ recirculate like other memory T cells (as reviewed by Di Rosa et al. [26]). Importantly, if such cells are not travelling to meet antigen, then antigen must be brought to them; in this respect circulating dendritic cells may play a pivotal role [28]. However, to date, the lifespan of bone marrow memory cells has not been quantified, nor has their contribution to the immune response following rechallenge. 

Even if such truly long-lived memory T cells do exist, a kinetic model is still appropriate as a paradigm for vaccine responses as the predominant pool of circulating memory cells is clearly in a state of dynamic equilibrium. In the next section we will consider what a kinetic model of a vaccine response might look like.

## 3. Part II—The Vaccine Response—A Kinetic Model

### 3.1. Thinking Phenotypically—Differentiation Pathways for Memory T Cells

**Figure 2:**
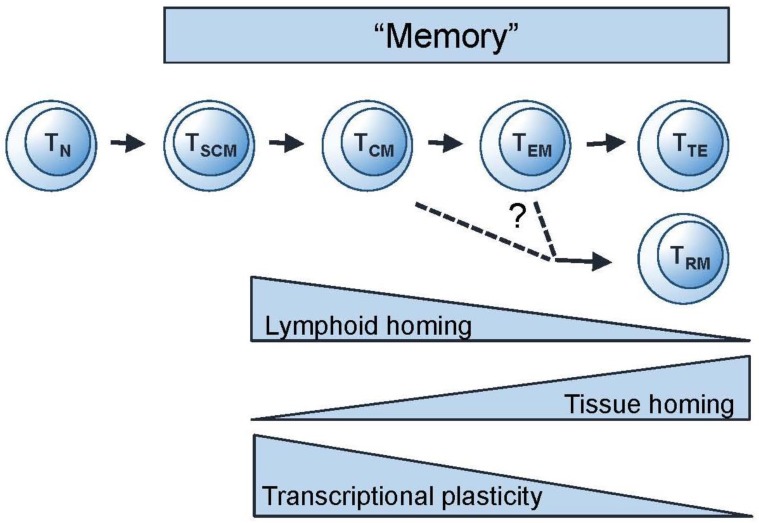
Linear differentiation model for T cells. Model for successive development of stem cell memory (T_SCM_), central memory (T_CM_), effector memory (T_EM_), and terminal effector memory (T_TE_) from naive (T_N_) T cells. The origin of T_RM_ is not yet fully clarified (see reference [29]). Adapted from Mahnke et al., 2013 [30] and Youngblood et al., 2015 [31].

**Figure 3:**
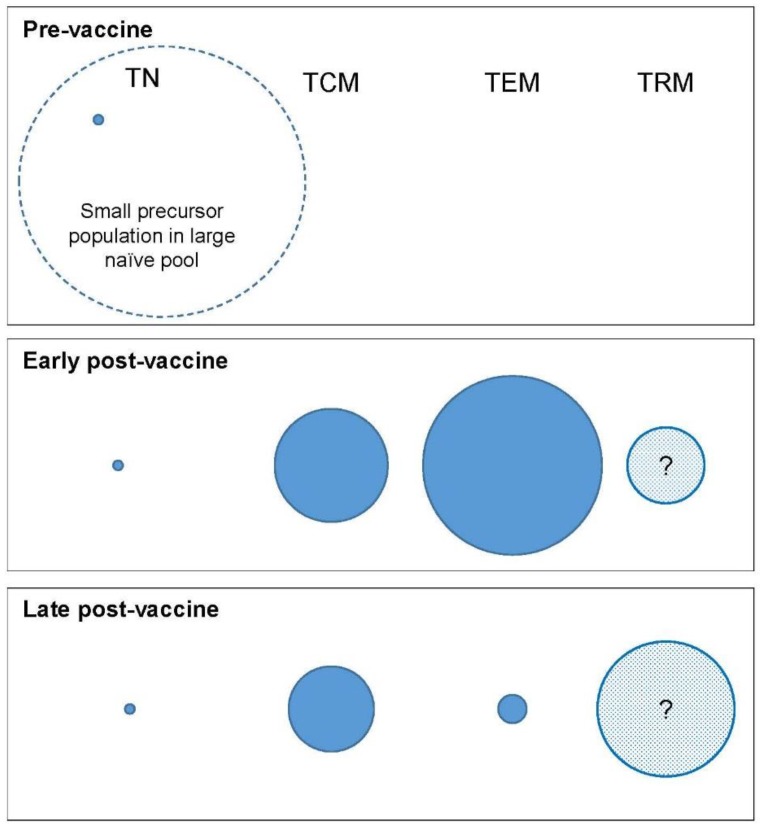
Compartment modelling in a vaccine response. Hypothetical simplified model of changes in compartment size following vaccination. The size of the circle represents schematically the relative number of cells of the corresponding phenotype—nave (T_N_), central memory (T_CM_), effector memory (T_EM_), and tissue-resident memory (T_RM_).

The conventional model for differentiation of both CD4^+^ and CD8^+^ T cell populations is a linear differentiation pathway in which cells progress along an immunological “one-way-street”; cells do not normally regress to a lower differentiation state (Figure 2) [30,32]. (Non-linear or alternative linear models have also been proposed [33] but are not discussed here; for full consideration see Restifo et al. [34]) A vaccine response, like a natural infection, would be expected to add to these pools in a sequential manner—i.e., T_SCM_ first, then central memory (T_CM_), through effector memory (T_EM_) to terminal effectors (T_TE_), including effector memory CD45 revertants (T_EMRA_—not shown in the figure) which are more likely to be formed with repeated restimulation. We can thus think about memory in phenotypic terms (Figure 3) and ask the questions: Which phenotypically-defined populations are expanded, which are contracted, and when? Since phenotype correlates with function, the phenotypic profile of the population will predict its functional attributes.

Three caveats merit consideration here: First, a semantic point: some authors (including this review) use the term “effector memory” [30], whereas others contrast “effectors” with “memory” cells [34], and others distinguish “primary effectors” from “secondary effectors” [35]. Second, for the purposes of discussion we have conflated CD4 and CD8 pools but it is self-evident that, although organised along broadly similar lines, significant differences exist between CD4 and CD8 memory. Thirdly, our analysis does not include “minor” populations, such as T follicular helper memory cells [35] which, although infrequent, may make a major functional contribution. 

**Figure 4:**
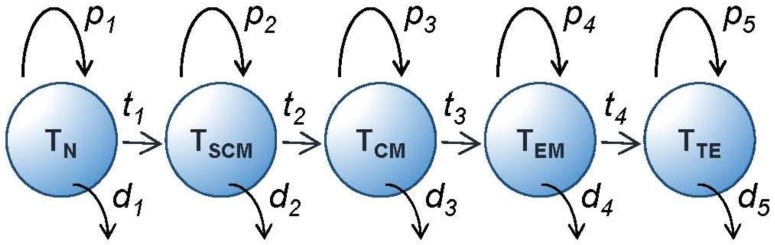
Kinetic model for T cell differentiation. Model of T cell memory population homeostasis. Circles represent phenotypically defined populations. Each population has a rate of intrinsic proliferation (*p*), a rate of transition to the next pool (*t*), and a rate of death (*d*). At equilibrium, all fluxes are in balance. Pool sizes are determined by the relative values of *p*, *d* and *t*. T_RM_ and other populations are not shown for simplicity in this diagram.

Bearing in mind these caveats, each phenotypically-defined subpopulation will have an average rate of turnover determined by the rate of entry of cells into the population and the rate of loss from the pool. The rate of entry will be the sum of two fluxes: the rate of proliferation within the pool plus the rate of entry into the pool by transition from other phenotypes (Figure 4). Similarly, the rate of loss will be the sum of the death rates of cells within the pool plus their transition rates out of the pool. At steady state, these will be equal. This model allows us to parameterise T cell homeostasis and analyse events from a kinetic viewpoint. During a vaccine response, dramatic inequalities between fluxes will result in massive expansion followed by contraction of phenotypically-defined subpopulations (Figure 3). Factors, such as local cytokine concentrations or ligand engagement, which modify these rates will therefore have a critical impact on the relative size of the different memory compartments preserved at the end of the acute vaccine response and this represents an opportunity to modulate the response.

Thinking about populations rather than individual cells in this way is important because it appears that there is considerable inter-cell heterogeneity, at least for CD8^+^ T cell responses. Gerlach et al. tracked the fate of individual naive CD8^+^ T cells in mice using DNA “barcoding” [36] and showed that both clonal and differentiation patterns are highly heterogeneous. Single naive cells generated either very large or very small families of progeny; furthermore, these progeny demonstrated considerable phenotypic variation, both within and between families. Although there was some evidence of fate imprinting, there was no evidence of asymmetric division as a driver for phenotypic diversity [36]. Thus, it appears that the predictability of T cell responses arises from population-averaging of a very diverse range of responses, rather than from uniformity at the single-cell level.

### 3.2. Thinking Clonotypically—Remodelling and Focussing

After the initial immune response, focussing occurs as a consequence of selection at the clonal level; some early clones die out rapidly whilst others appear late and persist. In primary human CMV infection, for example it has been shown by longitudinal clonotypic analysis that certain clones that were abundant in the primary response contracted disproportionately, whilst initially subdominant clones showed late expansion [37]. The pivotal determinant for selection into memory appeared to be the level of functional TCR avidity; high avidity clones being preserved whilst low avidity clones were lost [37]. The same clonal-focussing effect has also been reported in persistent murine polyoma virus-infection [38], and in a simian SHIV vaccination-rechallenge model [39], and is likely to represent a generic phenomenon.

### 3.3. Preservation of a Memory Population—Stochastic or Deterministic?

It is clear from the above discussion that signals that promote memory cell proliferation and survival must operate both within clones (primarily through TCR engagement) and across populations (primarily via cytokines and co-receptors) [31]. Such signals determine the relative likelihood of cell survival. It still remains the subject of debate whether the formation of a retained memory population is the stochastic consequence of random events happening heterogeneously across T cell populations [36], or results from the generation of a distinct population. The latter possibility is supported by work that suggests that “*cells destined to survive into the memory phase of the response can be identified at the effector stage, referred to as memory precursors*” [18]. Such cells manifest distinct transcriptional signatures and epigenetic changes. The existence of such a priori definable populations does not, however, exclude the possibility that they are generated stochastically rather than deterministically. 

## 4. Part III—Consequences of Thinking Kinetically about Vaccines

### 4.1. Rethinking the Target—Beyond ‘Bigger Is Better’

Considering vaccine responses in a dynamic context has several implications. Firstly, it can be seen from the above arguments that the primary goal of a vaccine is not to stimulate the largest possible number of acutely-responding cells, but rather to generate a long-lived population of high avidity, functional clones with an appropriate phenotypic distribution. Much work developing better adjuvants has focussed on increasing the size of the primary response, often used as a readout in vaccine studies, but indices of long-term memory may be a better index of success. Of course the two are not independent; larger primary responses tend to generate more memory cells [40], although the size of the primary response is also critically determined by the frequency of reactive cells in the naive repertoire [41]. 

It can also be seen from the dynamics of expansion and contraction that there may be greater opportunities to “improve” a vaccine by improving retention of memory cells rather than by increasing the magnitude of the initial response. For example, if one doubles the initial response, one may double the later memory population (assuming proportionality), but if one reduces the loss of cells between the acute response and the retained cell population by 2%, from 99.5% to 97.5%, the size of the final long-term memory population will be increased five-fold. Hence, retention/survival rates of memory cells may be more important to vaccine efficacy than the size of the primary response.

### 4.2. Focussing on Phenotype—and Beyond

A dynamic view also provides a framework within which optimal target phenotypic profiles for protection may be defined. These profiles may then be pursued experimentally. There is unlikely to be a single “best” pattern of response. Different pathogens may require different T cell phenotype profiles for optimal control—for some, a T_EM_-biased response will be required, whereas for others a T_SCM_ or T_CM_ biased response may be more favourable. For example, HIV protection may require a predominantly T_EM_ response [42] and this may be why CMV-based vectors, that provide continuous ongoing antigen-stimulation, result in control of infection with SIV [43]. Varying the strength, timing and duration of antigen exposure is one way to modify the balance between activation, memory, senescence and exhaustion [44]. Further epigenetic programming may occur even within cell populations that share a single phenotype; thus, repeated antigen exposure drives T_EM_ towards characteristic secondary, tertiary, and quaternary molecular signatures without further phenotype change [45]. The opportunity to intentionally programme cells to acquire and retain a specific phenotypic or molecular profile by the way a vaccine is delivered (dose, route, timing, etc.) represents an important advance in vaccinology.

### 4.3. Room for Change

A kinetic model also offers a basis for conceptualising how existing populations may be remodelled to accommodate new populations as they are added to the memory repertoire. Despite massive clonal expansions and contractions, the size of the human total lymphocyte pool remains remarkably constant throughout adult life. There are some global changes, of course; with advancing age, the naive T cell pool tends to contract in concert with falling thymic output, while the memory T cell pool expands slightly, but most new memory populations are accommodated within a constrained envelope. It has been suggested that, as a result of the constraint on total memory cell numbers, immunological memory for previously encountered antigens may be gradually lost when new memory cells are recruited, a phenomenon known as memory attrition. However, studies in mice [46] as well as recent investigations of acute human EBV infection, suggest that even dramatic acute CD8 T cell expansions can be accommodated without substantially depleting pre-existing memory for heterologous infections [47]. Moreover, recent studies have shown that the T_RM_ pool may be more flexible than the circulating pool of T_CM_ and T_EM_ [48].

If there is, in some sense, competition for “space” in the memory pool, then a dynamic system in which clones are maintained by homeostatic proliferation may result in better retention of memory. In a dynamic system, clone-size can change continually; hence where “space” is limited, the clone-size may reduce but the clone persists. Conversely, in a static system (like a library), populations are less plastic and the risk of losing the clone altogether may be greater. In view of their relatively short lifespan [13], circulating T_EM_ and T_CM_ populations are likely to be most flexible [46]. By contrast, the T_RM_ pool appears to be less constrained and this feature may be associated with greater longevity of individual T_RM_ cells [29]. 

### 4.4. Dynamic Systems Offer Opportunities for Modulation

One property of dynamic systems is that they offer opportunity for modulation; small changes in proliferation or death rates can result in large changes in T cell numbers. A good example is the successful use of immune checkpoint blockade of the costimulatory CD28 pathway; suppressing CD28 co-stimulation via CTLA-4 ligation limits the immune response by inducing T cell apoptosis. Conversely, activating CD28 prevents apoptosis and supports T cell expansion and differentiation [49,50]. Both effects have been harnessed therapeutically, for instance to prevent transplant rejection and to promote anti-tumour responses, respectively. Other strategies such as blockade of inhibitory receptors, interleukin-2 administration, regulatory T cell modulation, and targeting of mTOR, have all been suggested as potential tools to enhance CD8 T cell immunity [51]. IL-15 appears to have a particular key-role in determining the proliferation and survival rates of memory CD8^+^ T cells through an M-TOR dependent pathway [52]. In addition, knowledge of factors limiting the responsiveness of T cell memory populations may allow enhancement of memory responses. In the elderly, for example, it appears that P38 may be a crucial contributor to CD8 T cell senescence and that its effects may be reversible [53]. All these effects can be viewed through a kinetic prism by assessing whether their primary effects are on proliferation, phenotype-transition, function or death.

### 4.5. Thinking Locally—Directing the Immune Response

Although much of our knowledge of human T cell immunity is derived from and targeted towards circulating cells, it has become increasingly clear that local responses are pivotal in pathogen protection and clearance. Recent studies have suggested that there are opportunities to optimise vaccine efficacy by increasing immune protection at peripheral sites of pathogen entry [20,23,43]. Mucosal vaccination specifically has been advocated as a strategy to protect against mucosa-acquired infections such as herpes simplex, HIV, and influenza [54]. However, a dynamic model would emphasise that local populations not only have their own equilibrium, but also exist in dynamic equilibrium with systemic immune cells, hence the intriguing strategy of “prime and pull” in which a systemic prime is followed by recruitment of activated T cells to a local site. In a murine model, Shin and Iwasaki gave a conventional parenteral vaccination but followed it with local topical chemokine application given during the effector phase; this single chemokine “pull” was sufficient to establish a long-term population of tissue-resident memory CD8^+^ (but not CD4^+^) T cells within the vagina [55]. 

Generating T_RM_ may thus represent a specific target for future vaccine strategies. Interestingly, the effect of T_RM_ cells extends beyond their immediate availability and epitope-specific effects at the site of pathogen-entry; their presence also appears to create a "pathogen alert" state in the local tissue environment, which allows skin T_RM_ cells to contribute to protection against antigenically-unrelated viruses [56].

### 4.6. Implications for Viral Latency

Finally, if memory T cells are in fact short-lived cells, this may have implications for models of latency for viruses that reside in cells of the T cell lineage. For HIV infection, for example, there has been much debate over the location and size of the persistent viral reservoir. It is often stated that the long-lived viral reservoir resides in long-lived cells and strategies to activate those cells out of “latency” are being actively pursued. However, if a latent virus is passed on from cells of a dividing clone to their progeny, persistence may in fact result from persistence of the clone, rather than persistence of individual cells [57].

## 5. Conclusions

In this paper we have presented a kinetic view of T cell memory. Taking this view allows fundamental questions in human vaccinology to be addressed from a fresh perspective. Central to this approach is the appreciation that T cells operate as populations composed of diverse and heterogeneous individual cells—“memory” is a system property generated by the behaviour of clonal populations rather than a characteristic of individual cells. Of course some cells may be programmed to contribute more to memory than others [18,31], but the overall effect is achieved by dynamic populations.

For human vaccinology, many questions remain unresolved. Most of our knowledge on humans relates to circulating cells, because that is what we can sample; we need to know more about tissue-resident and bone-marrow-resident cells, particularly in terms of their kinetics and longevity. This is a field where animal data have led the way, but we need to know how best to use such animal data to inform human immunology, not just in terms of inter-species translation [58], but also in terms of real-world versus laboratory-environment. Memory responses are profoundly altered by life in a microbe-rich environment, as shown by differences between “clean” laboratory mice and those either living outside laboratories [59], or exposed to multiple pathogens [60]. We need to know if kinetic modelling can assist in the development of optimal vaccination schedules which have largely been based on empiric experimental data until now. One specific area of interest is the apparent need for ongoing antigenic stimulation for protective responses to HIV, as shown in simian models with live CMV-based vectors [42,43]. Might kinetic modelling allow us to hypothesise alternative approaches that do not require human “infection” with a CMV-like vaccine? Our understanding of human T cell memory has grown enormously through the characterisation of key phenotypic markers [30,32]; discovery of novel markers may further enhance our understanding of memory subsets. Finally, there is clearly much more to be learnt from epigenetics and transcriptomics, particularly in terms of lineage commitment [31,45].

Better understanding of the organisation and regulation of T cell responses will open up new opportunities for modulation and modification of vaccine responses. As Kamphorst et al. observed: “*Increased knowledge on the cellular and molecular requirements for CD8 T cell activation has unveiled new opportunities to directly modulate CD8 T cells to generate optimal responses.”* [51]. We hope that this analysis has demonstrated the value of viewing immune memory as a kinetically-based whole system-response.
